# Three-Quarters of Persons in the US Population Reporting a Clinical Diagnosis of Fibromyalgia Do Not Satisfy Fibromyalgia Criteria: The 2012 National Health Interview Survey

**DOI:** 10.1371/journal.pone.0157235

**Published:** 2016-06-09

**Authors:** Brian Walitt, Robert S. Katz, Martin J. Bergman, Frederick Wolfe

**Affiliations:** 1 Georgetown University, Washington, DC, United States of America; 2 Department of Rheumatology, Rush University Medical Center, Chicago, Illinois, United States of America; 3 Department of Rheumatology, Drexel University College of Medicine, Philadelphia, Pennsylvania United States of America; 4 National Data Bank for Rheumatic Diseases, Wichita, Kansas, United States of America; University of Würzburg, GERMANY

## Abstract

**Objectives:**

Although fibromyalgia criteria have been in effect for decades, little is known about how the fibromyalgia diagnosis is applied and understood by clinicians and patients. We used the National Health Interview Survey (NHIS) to determine the prevalence of self-reported clinician diagnosed fibromyalgia and then compared demographics, symptoms, disability and medical utilization measures of persons with a clinical diagnosis of fibromyalgia that did not meet diagnostic criteria (false-positive or prior [F/P] fibromyalgia) to persons with and without criteria-positive fibromyalgia.

**Methods:**

The National Health Interview Survey (NHIS) collected information about both clinical diagnosis and symptoms of fibromyalgia that was appropriately weighted to represent 225,726,257 US adults. Surrogate NHIS diagnostic criteria for fibromyalgia were developed based on the level of polysymptomatic distress (PSD) as characterized in the 2011 modified American College of Rheumatology criteria (ACR) for fibromyalgia. Persons with F/P fibromyalgia were compared with persons who do not have fibromyalgia and those meeting surrogate NHIS fibromyalgia criteria.

**Results:**

Of the 1.78% of persons reporting a clinical diagnosis, 73.5% did not meet NHIS fibromyalgia criteria. The prevalence of F/P fibromyalgia is 1.3%. F/P fibromyalgia is associated with a mild degree of polysymptomatic distress (NHIS PSD score 6.2) and characterized by frequent but not widespread pain and insomnia. Measures of work disability and medical utilization in F/P fibromyalgia were equal to that seen with NHIS criteria positive fibromyalgia and were 6-7x greater in F/P fibromyalgia than in non-fibromyalgia persons. F/P fibromyalgia was best predicted by being female (Odds Ratio [OR] 8.81), married (OR 3.27), and white (OR 1.96). In contrast, being a white, married woman was only modestly predictive of NHIS (criteria positive) fibromyalgia (OR 2.1).

**Conclusions:**

The majority of clinically diagnosed fibromyalgia cases in the US do not reach levels of severity necessary and sufficient for diagnosis. The clinical diagnosis of fibromyalgia is disproportionally dependent on demographic and social factors rather than the symptoms themselves. Diagnostic criteria for fibromyalgia appear to be used as a vague guide by clinicians and patients, and allow for substantial diagnostic expansion of fibromyalgia.

## Introduction

For nearly three decades, fibromyalgia has been an increasingly common diagnosis. In 2015, using the 2012 National Health Interview Survey (NHIS), we estimated that 1.75% (95% CI 1.42, 2.07), or 3.94 million US adults report severe levels of pain and somatic distress consistent with fibromyalgia diagnostic criteria [1]. Fibromyalgia is associated with billions of dollars in health care spending each year and with rates of work disability that approach 56% [2, 3]. Despite this substantial impact on public health, there is no evidence that clinicians generally use published fibromyalgia criteria for diagnosis, so that little is known about the validity of the diagnosis and how it is applied and understood by clinicians and patients.

Since its initial description, fibromyalgia has required the presence of chronic generalized pain and a particular set of symptoms of sufficient severity. The initial severity standard in 1990 was the presence of American College of Rheumatology (ACR) defined widespread pain and at least 11 of 18 predefined tender points on examination [4–6]. Richer clinical descriptions of fibromyalgia coupled with problems inherent in the measurement of tender points [7, 8] led to the development of the 2010 preliminary American College of Rheumatology criteria and their subsequent modification for survey research [9, 10] that introduced the polysymptomatic distress scale (PSD) as a severity measure for fibromyalgia. The PSD scale, which is also known as the *fibromyalgia severity scale*, transforms individual complaints regarding number of painful body areas and severity of fatigue, unrefreshing sleep, cognitive dysfunction, and other somatic and psychiatric symptoms into a single value ranging from 0–31. In epidemiological studies, PSD can be used to estimate the presence and symptom severity of fibromyalgia by using an appropriate cut point for diagnosis [2, 10].

Currently, the medical literature suggests that there is a substantial discordance between how the fibromyalgia diagnosis is defined and how it is applied in practice. Studies comprising thousands of patients derived from specialty clinics, general surveys, and pharmaceutical trials observe fibromyalgia to be a disorder nearly exclusively of middle-aged Caucasian women. Studies, derived mainly from Western Europe and the United States describe fibromyalgia patients typically being between 45–55 years old, over 90% Caucasian, and over 90% female [11–15]. However, population-based epidemiological studies using research criteria observe fibromyalgia to be a more equitable illness, with a modest female predominance of 2–3:1 and no important differences related to age or ethnicity [16–18]. Taken together, these data suggest that factors other than symptoms themselves play a critical role in the application and acceptance of the fibromyalgia diagnosis by clinicians and patients. We define this observation as “discordance”, the disparity between the *syndrome* of fibromyalgia, defined by the self-report of severe and specific fibromyalgia symptoms, and the *diagnosis* of fibromyalgia, defined by the receipt of the diagnosis from a medical professional. To determine without bias the nature of this discordance would require the use of a cohort that is designed to represent the general population and to collect data about fibromyalgia symptoms and clinical diagnostic status.

The National Health Interview Survey (NHIS) is a multi-purpose health survey conducted by the National Center for Health Statistics (NCHS), Centers for Disease Control and Prevention, and is the principal source of information on the health of the civilian noninstitutionalized household population of the United States [19]. It is designed to provide population-level estimates of point prevalence of health and health-related outcomes in the US population. Along with a wide array of demographic and health information, the 2012 NHIS collected information about adverse symptoms, including many that comprise elements of the 2010 ACR fibromyalgia criteria. We have recently demonstrated that 2012 NHIS questions can be used to approximate the modified 2010 ACR fibromyalgia criteria for epidemiological studies, allowing an estimate of the prevalence if fibromyalgia in the general population [10, 20]. The NHIS collected data about reported fibromyalgia diagnostic status for the first time in 2012. Thus, the 2012 National Health Interview Survey (NHIS) is a research population that possesses all of the necessary aforementioned qualities for studying the seeming discordance of fibromyalgia.

Here, we describe fibromyalgia in the general population, as both a criteria-based symptom construct (NHIS fibromyalgia) and as identified in the community (Clinical fibromyalgia). We then compare and contrast these different understandings of fibromyalgia and report the results below.

## Methods

### Subjects

We studied subjects from *The National Health Interview Survey (NHIS)*, a multi-purpose health survey conducted by the NCHS, Centers for Disease Control and Prevention.[19] The 2012 survey used a multi-stage clustered sample design, and over-sampled non-Hispanic black and Hispanic persons to allow for more accurate national estimates of health for these minority populations. The survey contains four main modules: Household, Family, Sample Child, and Sample Adult. The first two modules collect health and sociodemographic information on each member of all families residing within a sampled household. Within each family, additional information is collected from one randomly selected adult (the “sample adult”) aged 18 years or older. The overall 2012 response rate was 79.7%. Approximately one quarter of the NHIS adult sample were randomly selected to receive the Adult Functioning and Disability Supplement (AFD). In our analyses we merged the AFD dataset with other 2012 NHIS datasets according to NHIS instruction and weighting. The merged datasets assessed 8,446 individuals who represent a weighted population size of 225,726,257.

### Diagnostic Classification of Fibromyalgia and Polysymptomatic Distress

The NHIS questionnaires asked persons to provide certain information that is relevant to the fibromyalgia diagnosis. Two related NHIS “Adult Conditions Section (ACN)” questions inquired about whether persons had a clinical diagnosis of fibromyalgia:

ACN 290: Have you EVER been told by a doctor or other health professional that you have some form of arthritis, rheumatoid arthritis, gout, lupus, or fibromyalgia (fy-bro-my-AL-jee-uh)?

ACN 297: You just mentioned that you were told by a doctor or other health professional that you had some form of arthritis, rheumatoid arthritis, gout, lupus, or fibromyalgia (fy-bro-my-AL-jee-uh). Which of these were you told you had?

Persons that answered affirmatively on ACN 290 and “fibromyalgia” on ACN 297 were classified as having current or previous *clinical fibromyalgia*; all other persons were classified as not having a clinical fibromyalgia diagnosis.

### Surrogate criteria based criteria for fibromyalgia

The preliminary 2010 American College of Rheumatology criteria is a current standard for determining the diagnosis of fibromyalgia [9, 10], and its 2011 modification provides a mechanism for survey research. To meet the 2011 modified ACR criteria requires the reporting of sufficiently high levels of widespread pain, fatigue, sleep, and cognitive problems. However, these exact questionnaire variables are not available in the 2012 NHIS. Therefore we developed surrogate fibromyalgia diagnostic criteria and polysymptomatic distress (PSD) scores for the NHIS using a questionnaire that contained both modified ACR questions and similar NHIS variables that evaluated joints, regional pain sites, and fatigue, sleep, and cognitive complaints. The complete validation methodology has been described in detail previously [2]. In brief, we administered the combined questionnaire to 415 rheumatic disease patients, including those with fibromyalgia, in two clinical rheumatology practices during ordinary clinical care. Using multivariable regression analysis, we developed a model by regressing the modified ACR PSD score on selected NHIS variables. The area under the receiver operating curve (AUC ROC) of the model was 94.6%. 88.1% of cases were properly classified, and the sensitivity/specificity was 74.7%/93.4%. Ten-fold validation with 100 replications showed a ROC of 90.1% (95% CI 89.7, 90.2). Based on the rheumatology clinic study analyses and prior data, we selected a PSD score of ≥13 to designate a *fibromyalgia case* (NHIS FM+). At this PSD level, 85.5% satisfied the ACR 1990 criteria definition of widespread pain [5]. In the recent German population study of 2,445 subjects, 82.7% of fibromyalgia positive participants had widespread pain [18]. Persons in the NHIS with PSD scores ≤ 12 were considered not to have fibromyalgia (NHIS FM-). To be clear, the PSD score used in the NHIS data analyses was the score developed with the rheumatology clinic data. References to PSD not in the NHIS refer to the score directly calculated from the modified ACR 2010 fibromyalgia criteria.

The NHIS definition found to best approximate 2010 ACR criteria included: specific multiple joint sites (right and left hand/wrist, elbow, shoulder, hip, and knee), unpaired sites of low back pain, face pain and abdominal pain, and tiredness (fatigue) and concentration ability. As we have previously reported, the NHIS criteria and the 2010 ACR criteria differ in several ways [2]. The ACR criteria inquired about broad, predominately non-articular areas while the NHIS questionnaires directed attention to specific joint regions. Symptom variables differed in their wording and severity, and there were fewer germane symptom variables available in the NHIS data. The NHIS PSD score had a shorter range than the original PSD, perhaps related to these overall differences. Despite these limitations, the application of NHIS fibromyalgia criteria and NHIS PSD scoring result in very similar fibromyalgia prevalence and PSD scoring to that seen in the German general population study that utilized the 2010 modified ACR criteria [18]. We believe the data are useful and appropriate surrogates when interpreted with appropriate degrees of uncertainty.

### Diagnostic Classification

To explore the relation between a clinical diagnosis of fibromyalgia and NHIS fibromyalgia criteria, we divided the NHIS cohort into 3 diagnostic categories: (1) Not Fibromyalgia: Persons were considered not to have fibromyalgia if they did not self-report having a clinical fibromyalgia diagnosis and did not meet NHIS fibromyalgia criteria. (2) False-Positive or prior (F/P) Fibromyalgia: For ease of presentation, we labeled persons who self-reported a clinical fibromyalgia diagnosis but did not meet NHIS study criteria as F/P fibromyalgia.” (3) NHIS Fibromyalgia: This category included all persons that met specific NHIS fibromyalgia criteria. Persons with and without a clinical diagnosis of fibromyalgia were both included in this group.

We consider persons to be in the F/P category if they fail to satisfy NHIS criteria. In this sense, we are examining “point prevalence” and we are following the rule of all prior epidemiological studies and clinical trials by diagnosing fibromyalgia only when recognized, published criteria are met. Some patients who have been previously clinically diagnosed with fibromyalgia, however, may improve symptomatically and therefore fail at a follow-up time to meet test criteria. Because the NHIS survey does not attach a time frame to the question, “Have you EVER been told by a doctor or other health professional that you had … fibromyalgia,” patients that we have labeled F/P might also include those previously correctly diagnosed.

### Statistical analyses

Statistical analyses were performed using Stata version 13.1 [21]. NHIS analyses incorporated appropriate stratification and weights as specified by the NHIS survey design. Odds ratios were calculated using unadjusted and adjusted logistic regressions as noted. Population proportions were obtained using Stata’s margins procedure. The presence of stepwise differences in proportions across multiple groups was obtained with Stata’s ologit procedure.

## Results

### Prevalence of the clinical diagnosis of fibromyalgia and F/P fibromyalgia

The 2012 NHIS survey evaluated 8,446 persons weighted to represent 225,536,654 persons in the US general population. Of these, 1.78% of population reported being told by a physician or health professional that they had fibromyalgia and 1.75% persons surveyed met NHIS fibromyalgia criteria (Table 1). When applied to persons with a clinical diagnosis of fibromyalgia, 73.5% did not have fibromyalgia by NHIS criteria (Table 1). The NHIS survey estimates the overall prevalence of F/P fibromyalgia to be 1.3% in the US population, a percentage that represents 2,955,897 persons.

**Table 1:** Fibromyalgia status according to physician diagnosis (Column 1) and NHIS criteria (Top 3 rows) and NHIS criteria (Column 1) and physician diagnosis (Bottom 3 rows) in 2012 National Health Interview Survey.

Category	% NHIS FM -	% NHIS FM +	% All Subjects
MD FM-	98.7	1.3	98.2
MD FM +	73.5	26.6	1.8
Total	98.3	1.7	100.0
	% MD FM -	% MD FM +	
NHIS FM-	98.7	73.0	98.3
NHIS FM +	1.3	27.0	1.7
Total	98.3	1.7	100.0

MD FM -: Persons that do not report receiving a fibromyalgia diagnosis from a physician or health professional. MD FM+: Persons that do report receiving a fibromyalgia diagnosis from a physician or health professional. NHIS FM -: Persons that do not satisfy 2012 NHIS fibromyalgia criteria. NHIS FM+: Persons that do satisfy 2012 NHIS fibromyalgia criteria.

### The symptoms of F/P fibromyalgia are different from those based on fibromyalgia criteria

The reporting of symptoms in F/P fibromyalgia is compared to that of not fibromyalgia and NHIS fibromyalgia persons in Table 2. Column 5 compares not fibromyalgia persons to F/P fibromyalgia; column 6 compares F/P fibromyalgia to NHIS fibromyalgia persons. F/P fibromyalgia pain was best characterized by being frequent (77.5%), involving low back (67.5%), but not being particularly widespread (29.2%). Persons with F/P fibromyalgia on average experienced fatigue more than 3 days a year (57.3%), that was not typically severe (29.8%), and insomnia (54.1%). Psychological symptoms were not uncommon, with a 3 fold increases in self-reported anxiety in the last three months (33.3%) and 1.8 fold increase in having depression ever (41.2%) compared with persons not having fibromyalgia.

**Table 2:** Comparison of somatic and psychological symptoms between persons without fibromyalgia, FP fibromyalgia, and those that meet NHIS fibromyalgia criteria.

Symptom Profile	*1*: *Not fibromyalgia*	*2*: *F/P Fibromyalgia*	*3*: *Fibromyalgia*	*2 vs. 1 p-value†*	*2 vs. 3 p-value†*
Percent in category (%)	96.9	1.3	1.7		
	Mean (SE)	Mean (SE)	Mean (SE)		
Polysymptomatic Distress Score	2.5 (0.04)	6.8 (0.36)	16.1 (0.32)	<0.001, <0.001	<0.001,<0.001
	Pct. (SE)	Pct. (SE)	Pct. (SE)		
Widespread Pain (%)	4.9 (0.3)	29.2 (4.7)	83.6 (3.4)	<0.001, <0.001	<0.001,<0.001
Pain: most or all days (%)	15.2 (0.5)	77.5 (4.6)	86.6 (3.9)	<0.001, <0.001	0.126, 0.236
Fatigue more than 3 days last year (%)	12.8 (0.5)	57.2 (5.3)	81.3 (4.4)	<0.001, <0.001	<0.001, 0.002
Severe Fatigue (%)	9.5 (0.4)	29.8 (5.0)	59.6 (5.1)	<0.001, <0.001	<0.001,<0.001
Insomnia past year (%)	18.2 (0.5)	54.1 (5.5)	67.5 (5.0)	<0.001, <0.001	0.078, 0.150
Memory loss past year (%)	3.9 (0.2)	9.1 (3.0)	43.6 (5.5)	0.090, 0.172	<0.001,<0.001
Low back pain past 3 months (%)	26.7 (0.6)	67.5 (5.1)	94.2 (2.1)	<0.001, <0.001	<0.001,<0.001
Abdominal pain past 3 months (%)	7.8 (0.3)	19.7 (4.4)	66.6 (4.6)	0.008, 0.015	<0.001,<0.001
Migraine past 3 months (%)	13.2 (0.5)	33.6 (5.3)	56.2 (5.6)	<0.001, <0.001	0.004, 0.008
Allergies past year (%)	17.8 (0.6)	17.9 (4.0)	49.7 (4.8)	0.981, 0.999	<0.001,<0.001
Depression Ever (%)	13.0 (0.4)	41.2 (5.7)	62.7 (5.2)	<0.001, <0.001	0.006, 0.012
Anxiety past 3 months (%)	17.9 (0.5)	33.3 (4.6)	64.8 (5.2)	0.001, 0.002	<0.001, 0.001
Phobias Ever (%)	4.6 (0.3)	13.9 (4.6)	32.9 (4.4)	0.044, 0.087	0.003, 0.006

Not fibromyalgia: Persons that do not report receiving a fibromyalgia diagnosis from a physician or health professional and do not satisfy 2012 NHIS fibromyalgia criteria. F/P fibromyalgia: Persons that do report receiving a fibromyalgia diagnosis from a physician or health professional and do not satisfy 2012 NHIS fibromyalgia criteria. Fibromyalgia: Persons that do satisfy 2012 NHIS fibromyalgia criteria.

†: First p-value is unadjusted, second p-value is adjusted for multiple comparisons by Sidak’s method

Compared with NHIS fibromyalgia, F/P fibromyalgia was considerably milder. Persons with F/P fibromyalgia had a mean PSD score of 6.2 (95% CI: 5.5–6.9), which is considered a “mild level” of PSD [22] and 2.3x less than what is seen in NHIS fibromyalgia–which requires as PSD score of at least 13. NHIS fibromyalgia persons were more likely to have widespread pain (2.9x), severe fatigue (2x), memory loss in the last year (4.8x), abdominal pain (3.4x), migraines (1.7x), depression (1.5x), and anxiety (1.9x) than that seen in F/P fibromyalgia. In summary, persons with F/P fibromyalgia report frequent pain and insomnia but do not frequently report widespread pain nor many of the somatic complaints that comprise ACR and modified ACR criteria or OMERACT key symptom domains [6, 9].

### The impact of F/P fibromyalgia is equal to NHIS fibromyalgia on disability and medical utilization measures

Table 3 details the relation between F/P fibromyalgia and measures of work disablement and medical utilization. In persons under 65, satisfying F/P fibromyalgia criteria had a substantial impact on disability measures. Compared with persons without fibromyalgia, F/P fibromyalgia persons were more likely to apply for disability (7.2x), receiving disability (5.9x), and being unable to work for health reasons (6.5x). In contrast, no statistical differences were seen between F/P fibromyalgia and NHIS fibromyalgia persons under 65 in disability applications or being unable to work due to health concerns. The two-fold comparative increase in receiving disability in the last year seen in the NHIS fibromyalgia group may reflect the importance of symptom severity in meeting Social Security Disability Insurance criteria.

**Table 3:** Comparison of selected work and health utilization between persons without fibromyalgia, FP fibromyalgia, and those that meet NHIS fibromyalgia criteria.

Variable	1:Not fibromyalgia	2:F/P Fibromyalgia	3: Fibromyalgia	2 vs. 1 p-value†	2 vs. 3 p-value†
Work/disability*					
	Pct. (SE)	Pct. (SE)	Pct. (SE)		
Applied for Disability (Ever) (%)	5.4 (0.3)	36.1 (6.4)	46.2 (6.6)	<0.001, <0.001	0.293, 0.500
Received Disability Last Year (%)	2.6 (0.2)	15.3 (3.7)	30.5 (5.1)	<0.001, 0.002	0.016, 0.032
Unable to work due to health (%)	5.4 (0.3)	35.0 (6.6)	51.4 (6.4)	<0.001, <0.001	0.087, 0.167
*Medical Utilization*					
	Pct. (SE)	Pct. (SE)	Pct. (SE)		
Specialist Visit in Year (%)	24.6 (0.6)	60.1 (5.2)	55.9 (4.7)	<0.001, <0.001	0.544, 0.792
Generalist Visit in Year (%)	65.9 (0.6)	87.4 (3.2)	78.9 (3.8)	<0.001, <0.001	0.082, 0.157
Hospitalized in year (%)	8.7 (0.4)	12.9 (3.8)	18.6 (3.0)	0.278, 0.479	0.247, 0.432
Depression medication (%)	7.7 (0.3)	36.1 (5.7)	44.7 (0.5)	<0.001, <0.001	0.252, 0.441
Anxiety medication (%)	8.2 (0.3)	35.9 (5.7)	43.6 (4.5)	<0.001, <0.001	0.311, 0.525

Not fibromyalgia: Persons that do not report receiving a fibromyalgia diagnosis from a physician or health professional and do not satisfy 2012 NHIS fibromyalgia criteria. F/P fibromyalgia: Persons that do report receiving a fibromyalgia diagnosis from a physician or health professional and do not satisfy 2012 NHIS fibromyalgia criteria. Fibromyalgia: Persons that do satisfy 2012 NHIS fibromyalgia criteria.

Work/disability: Variables concerning health-related ability and work disablement. Medical Utilization: Variables concerning use of health-related resources

* <age 65.

†: First p-value is unadjusted, second p-value is adjusted for multiple comparisons by Sidak’s method

F/P fibromyalgia persons report substantial increases in medical utilization compared with non fibromyalgia persons. They are 1.3x more likely to see a general practitioner and 2.4x more likely to see a medical specialist than non fibromyalgia persons, equivalent to what is seen in NHIS fibromyalgia persons. They are also more likely to use antidepressants (4.7x) and anxiolytics (4.4x) than persons without fibromyalgia. The rates of self-reported antidepressant and anxiolytic use in F/P fibromyalgia are equal to what is seen in NHIS fibromyalgia, despite the statistically significant differences in depression and anxiety reporting detailed above. No differences in self-reported hospitalizations were seen between F/P fibromyalgia and the other categories.

### Demographics and Lifestyle Factors Predict Diagnosis with Fibromyalgia

The comparative influence of essential demographic and lifestyle factors on F/P fibromyalgia is shown in Table 4. Persons with F/P fibromyalgia were almost always women (92.7%) and married (74.6%) compared with non fibromyalgia persons (50.8%, p<0.001; 51.7%, p<0.001) and NHIS fibromyalgia persons (70.7%, p<0.001; 43.3%, p<0.001). They were also more often white (82.2%) compared with persons who did not have fibromyalgia (66.9%, p<0.001), and a greater proportion with F/P fibromyalgia were white than in persons with NHIS fibromyalgia (p = 0.113). This finding did not reach significance, reflecting in part that NHIS fibromyalgia persons who were also diagnosed with fibromyalgia were also almost all white (90.8%). Only 66% of NHIS fibromyalgia persons without a physician diagnosis were white, which is equivalent to the proportion of white persons in the US population.

**Table 4:** Comparison of demographics and allied Factors between persons without fibromyalgia, F/P fibromyalgia, and those that meet NHIS fibromyalgia criteria.

Variable	1:Not fibromyalgia	2:F/P Fibromyalgia	3: Fibromyalgia	2 vs. 1 P-value†	2 vs. 3 P-value†
*Demographics*					
	Mean (SE)	Mean (SE)	Mean (SE)		
Age in years	46.4 (0.2)	53.2 (1.5)	51.4 (1.6)	<0.001, <0.001	0.370,0.603
	Pct. (SE)	Pct. (SE)	Pct. (SE)		
Sex (female) (%)	50.8 (0.7)	92.7 (0.3)	70.7 (0.4)	<0.001, <0.001	<0.001,<0.001
White (%)	66.9 (0.8)	82.2 (3.8)	73.5 (3.7)	<0.001, <0.001	0.102, 0.194
Black (%)	11.8 (0.5)	9.3 (2.7)	12.6 (2.9)	0.347, 0.574	0.399, 0.639
Hispanic (%)	15.2 (0.6)	5.8 (2.4)	10.5 (2.4)	<0.001, <0.001	0.190, 0.343
*Lifestyle Factors*					
	Pct. (SE)	Pct. (SE)	Pct. (SE)		
Married/Cohabitating (%)	51.7 (0.7)	74.6 (4.2)	43.3 (4.5)	<0.001, <0.001	<0.001, <0.001
Never Married (%)	22.6 (0.6)	4.4 (1.7)	17.5 (3.6)	<0.001, <0.001	0.001, 0.002
Divorced (%)	10.9 (0.4)	11.1 (3.0)	22.9 (3.5)	0.932, 0.995	0.011, 0.021
Not College Graduate (%)	71.7 (0.7)	81.9 (4.2)	87.5 (2.8)	0.018, 0.035	0.241, 0.424
W.H.O. Obese (%)	29.8 (0.7)	35.9 (5.0)	46.8 (5.0)	0.227, 0.402	0.152, 0.281
Smoking now (%)	18.6 (0.6)	25.8 (4.8)	38.5 (4.9)	0.139, 0.258	0.056, 0.109

Not fibromyalgia: Persons that do not report receiving a fibromyalgia diagnosis from a physician or health professional and do not satisfy 2012 NHIS fibromyalgia criteria. F/P fibromyalgia: Persons that do report receiving a fibromyalgia diagnosis from a physician or health professional and do not satisfy 2012 NHIS fibromyalgia criteria. Fibromyalgia: Persons that do satisfy 2012 NHIS fibromyalgia criteria.

W.H.O. Obese: World Health Organization obesity (Body Mass Index ≥ 30)

†: First p-value is unadjusted, second p-value is adjusted for multiple comparisons by Sidak’s method

Persons with F/P fibromyalgia were older and less educated than those without fibromyalgia, and similar to what was reported by NHIS fibromyalgia persons. Of interest, obesity and current smoking status were statistically significantly different between not fibromyalgia and NHIS fibromyalgia persons (p<0.001). F/P fibromyalgia persons reported levels of obesity and smoking that fell between the two aforementioned categories. No differences in total reported income was noted between the three diagnostic categories.

### Predicting the Fibromyalgia Diagnosis

**Fig 1:**
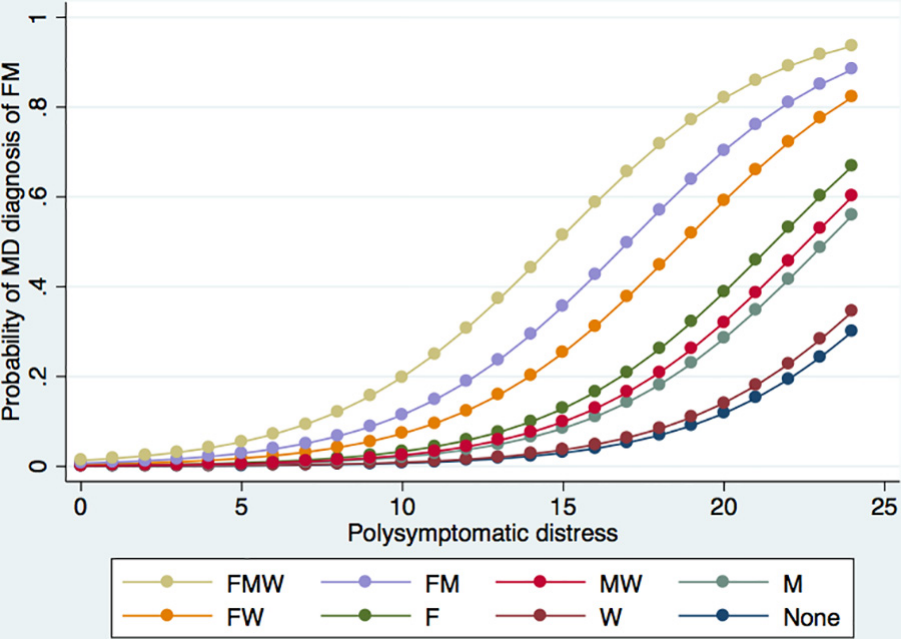
Probability of a person being told by a physician that they have fibromyalgia according to gender, ethnicity, and marital status. Codes are F (Female), M (Married), W (White) and None (Not female, married, or white). Groups are mutually exclusive.

To explain the wide array of findings reported above, we used the full dataset to develop regression models for predicting a clinical diagnosis of fibromyalgia and compared the results with similar regression models for NHIS fibromyalgia. Table 5 contains an explanatory model of the clinical fibromyalgia diagnosis. The strongest predictor is PSD 1.3, p<0.001). Holding PSD constant in the model, 3 demographic factors dominate physician diagnosis: being female (OR 8.8), being married (OR 3.3) and being white (OR 2.0). The odds ratio for a clinical diagnosis for the linear combination of all 3 present simultaneously is 56.3 (p<0.001), for female and married 28.8 (p<0.001) and for female and white 17.2 (p<0.001). When PSD is removed from the model (Table 6), the F value decreases as expected, and obesity (OR: 1.5), smoking (OR: 2.0), and having less than college education (OR: 1.8) become statistically significant, which indicates that these factors are subsumed by PSD. The additive effect of these three demographic variables across the range of PSD scoring is shown in Fig 1. Being white, being a woman, and being married influence the clinical fibromyalgia diagnosis meaningfully at levels of PSD as low as 5. In contrast, the probability of receiving a fibromyalgia diagnosis at a PSD score of 13 (the threshold for meeting NHIS fibromyalgia criteria) if a person is not a white, married woman is negligible.

**Table 5:** Multivariable logistic regression of predictors of physician diagnosis of fibromyalgia: Model predicting persons told by a physician that they that fibromyalgia (including Polysymptomatic Distress Score).

Variable	OR (95% Confidence Intervals)	t	P-value
Female	8.81 (4.48, 17.33)	6.32	<0.001
Married or cohabiting	3.27 (2.00, 5.34)	4.73	<0.001
White	1.96 (1.20, 3.20)	2.68	0.008
Age (years)	1.01 (1.00, 1.02)	2.00	0.046
W.H.O. Obese	1.03 (0.68, 1.57)	0.15	0.885
Current Smoker	1.10 (0.62, 1.94)	0.32	0.747
Less than College education	1.29 (0.72, 2.33)	0.86	0.392
Polysymptomatic Distress score	1.34 (1.28, 1.40)	13.67	<0.001
Female + married + white	56.30 (21.69, 146.11)	8.32	<0.001
Female + married	28.77 (12.35, 67.01)	7.82	<0.001
Female + white	17.23 (7.68, 38.71)	6.93	<0.001

**Table 6:** Multivariable logistic regression of predictors of physician diagnosis of fibromyalgia: Model predicting persons told by a physician that they that fibromyalgia (excluding Polysymptomatic Distress Score).

Variable	OR (95% Confidence Intervals)	t	P-value
Female	9.00 (4.79, 16.90)	6.87	<0.001
Married or cohabiting	2.2 (1.48, 3.30)	3.91	<0.001
White	2.2 (1.41, 3.47)	3.49	0.001
Age (years)	1.01 (1.00, 1.02)	3.70	<0.001
W.H.O. Obese	1.48 (1.02, 2.14)	2.07	0.039
Current Smoker	2.03 (1.26, 3.27)	2.91	0.004
Less than College education	1.75 (1.03, 2.99)	2.06	0.040
Female + married + white	44.2 (18.55, 105.30)	8.59	<0.001
Female + married	19.9 (9.37, 42.48)	7.79	<0.001
Female + white	20.0 (9.50, 41.93)	7.93	<0.001

We then modeled these same variables to determine their explanatory power for having NHIS fibromyalgia (Table 7). PSD is omitted, as it is a perfect predictor. Being female, married, or white are much less important predictors (OR for all 3 present simultaneously: 2.1, p = 0.039), indicating these factors have substantially less influence on how persons report having fibromyalgia symptoms compared to how persons report receiving a fibromyalgia diagnosis from physicians and other health professionals.

**Table 7:** Multivariable logistic regression of predictors of physician diagnosis of fibromyalgia: Model predicting persons meeting 2012 NHIS fibromyalgia criteria (excluding Polysymptomatic Distress Score).

Variable	OR (95% Confidence Intervals)	t	P-value
Female	2.24 (1.49, 3.38)	3.89	<0.001
Married or cohabiting	0.75 (0.51, 1.09)	-1.53	0.126
White	1.28 (0.86, 1.91)	1.22	0.222
Age (years)	1.02 (1.01, 1.03)	3.81	<0.001
W.H.O. Obese	1.99 (1.32, 3.01)	3.30	0.001
Current Smoker	2.87 (1.92, 4.29)	5.14	<0.001
Less than College education	2.08 (1.21, 3.56)	2.68	0.008
Female + married + white	2.14 (1.04, 4.43)	2.07	0.039
Female + married	1.67 (0.97, 2.87)	1.87	0.062
Female + white	2.88 (1.58, 5.23)	3.48	0.001

W.H.O. Obese: World Health Organization obesity (Body Mass Index ≥ 30)

## Discussion

The above results provide a unique insight into the nature of fibromyalgia in the community. Unlike previous clinical studies, the 2012 NHIS study is able to describe types of adverse symptoms and their severity without being confounded by health-care seeking behavior and clinical selection [1, 23]. In addition, the 2012 NHIS study is able to characterize how the fibromyalgia diagnostic label is applied in the unselected US population. Our findings substantiate the discordance in fibromyalgia characteristics observed in epidemiologic and clinical studies. Persons who are classified by criteria with the *syndrome* of fibromyalgia are not the same persons reporting a *diagnosis* of fibromyalgia. What emerges from this analysis is that these are two different understandings of fibromyalgia that, for the most part, do not overlap.

The first understanding is that the severe polysymptomatic distress that defines criteria positive fibromyalgia occurs in 1.75% of the population (~3.9 million persons), and is relatively equitable in terms of demographics. Meeting fibromyalgia criteria is not particular to the middle-aged nor ethnic group, and associated with a more modest gender disparity of 2.3:1 [2]. Social disadvantage appears to be more important than demographics, as evidenced by increased odds ratios of NHIS fibromyalgia in the divorced (OR:2.0, p<0.001) and non-college graduates (OR:1.4, p<0.001) [2]. Fibromyalgia-level symptomatology is experienced by more than 3.9 million persons in the US population, but only 27% of those persons report a clinical diagnosis. It is clear that having the severe adverse symptoms that define fibromyalgia is not essential to receiving the fibromyalgia diagnosis. Rather, many persons who satisfied NHIS criteria for fibromyalgia reported receiving alternative diagnoses, such as rheumatoid arthritis (15.3%), gout (3.3%), lupus (1.4%), low back pain (21.7%), and non-specific “arthritis” (47.5%) [2]. The same constellation of severe symptoms can be clinically interpreted in many different ways, perhaps influenced by clinician and patient beliefs and their resultant interactions. Published diagnostic criteria appear to be used only as a vague guide in determining what fibromyalgia is in clinical practice.

The second understanding is that fibromyalgia in the community, as it is diagnosed by clinicians and acknowledged by patients, represents something else entirely, as the majority of clinical (physician) fibromyalgia cases do not reflect severe polysymptomatic distress sufficient for criteria based diagnosis. Of persons reporting a clinical fibromyalgia diagnosis, 73.5% fail to meet NHIS criteria. However, persons with clinical fibromyalgia are not just the “worried well” [24]. They are typically symptomatic, often with frequent, local pain complaints, back pain, insomnia, anxiety, and depression at mild to moderate levels. There is also little evidence that clinical fibromyalgia represents what has been called a somatic symptoms disorder [25]. It is not disproportionate, excessive, or unreasonable that persons with mild to moderate amounts of polysymptomatic distress would seek medical advice about their symptoms.

We have used the term F/P fibromyalgia to classify a group of subjects who had a clinical diagnosis of fibromyalgia but did not satisfy NHIS criteria. F/P fibromyalgia represents both persons with a false-positive diagnosis and those who had prior fibromyalgia and achieved a relative remission in symptoms. Prospective study of the natural history and treatment of fibromyalgia suggests that the majority of F/P fibromyalgia represents a false positive diagnosis. Treatment of fibromyalgia is notoriously difficult [26]. Clinical trials demonstrate that only 1 in 11–12 persons has substantial benefit from modern pharmaceutical agents [13, 27]. Data from the American College of Rheumatology 2010 criteria study found that only 25% of 263 patients with a previous expert diagnosis of fibromyalgia did not satisfy 1990 criteria when examined for study inclusion [9]. A population-based study of 1,555 fibromyalgia patients also showed that only 25% of the population had moderate improvement in symptoms over a mean duration of 4 years [28]. Even if it was estimated that half of F/P persons were prior cases that symptomatically improved, the NHIS still estimates that 2 million fibromyalgia diagnoses in the US are false-positive.

This demonstration that the fibromyalgia diagnosis is frequently applied to persons with milder somatic complaints should raise serious concerns about medicalization and diagnostic expansion. In practice, well-intentioned clinicians apply the twin tools of diagnosis and treatment to patients to empathize, to palliate symptoms, and ensure societal approbation. However, the use of medical labels to define adverse but normal human problems is often not necessarily a harmless act. The NHIS data demonstrate that, despite milder symptoms, persons with clinical fibromyalgia have high levels of disablement (35%) and psychotropic medication use (36%). These levels are higher than seen in undiagnosed persons with comparable levels of PSD and they approach what is seen in persons meeting NHIS criteria. As diagnostic labeling can in itself encourage disablement [29] and indiscriminate prescription of pain and psychotropic medications can lead to injury and death [15, 30], the widespread use of the fibromyalgia diagnosis as an explanation of mild-to-moderate polysymptomatic distress has likely led to substantial harm and societal costs [31]. The data of this study make clear that the use of the fibromyalgia diagnosis has been expanded beyond the initial intent of the fibromyalgia diagnosis. This expansion appears fueled by programs to increase clinical awareness, through educational activities for clinicians, academic research, patient advocacy, and direct-to-patient advertising, much of which has been financed by the pharmaceutical industry [32]. Our data suggest that the term fibromyalgia has no clear valid or reliable clinical meaning or understanding; and is socially constructed.

It is important to recognize that the increased probability of being a married, white woman is essential to understanding fibromyalgia as a clinical disorder. Modern medicine has empowered patients to an unprecedented degree with vast increases in patient access to medical information and with the ability to be more selective about the diagnoses and treatments they will accept [33]. Fibromyalgia patients often take an active role in their diagnosis, recognizing their polysymptomatic distress as fibromyalgia and seeking clinical care for confirmation and treatment. With the patient-clinician relationship increasingly defined by partnership, it becomes less clear of what a “self-reported clinical diagnosis” of fibromyalgia is. We suggest the term best reflects persons for whom fibromyalgia has become part of their health narrative. Self-identification may have an important role in determining the clinical assignment of the fibromyalgia diagnosis.

## Conclusion

The majority of clinical fibromyalgia cases in the US do not reach levels of severity considered to be diagnostic. Despite relatively milder symptoms, clinical fibromyalgia is associated with high levels of work disability and medical utilization. Clinical fibromyalgia is disproportionally dependent on socially-constructed factors rather than the symptoms themselves. Diagnostic criteria appear to be used only as vague guide by clinicians and patients, allowing for substantial diagnostic expansion of fibromyalgia.

## Supporting Information

S1 File combnhis.dta.zip. The data file used for analysis in this study in zip format.
(ZIP)

S2 File Stata Commands Supporting File.docx. A file to assist interested persons in accessing the combnhis.dta.zip file.
(DOCX)
