# Supplementary material for: Three-Quarters of Persons in the US Population Reporting a Clinical Diagnosis of Fibromyalgia Do Not Satisfy Fibromyalgia Criteria: The 2012 National Health Interview Survey
Source: PLoS One. 2016 Jun 9;11(6):e0157235. doi: 10.1371/journal.pone.0157235 (PMC4900652; doi:10.1371/journal.pone.0157235)
Supplement: S2 File — A file to assist interested persons in accessing the combnhis.dta.zip file. (DOCX) [file pone.0157235.s002.docx]

The data file is in Stata Format

To access the data file, issue the following commands:

use combnhis, clear

set more off

svyset [pweight=wtfa_afd],strata(strat_p) psu(psu_p) singleunit(centered)

keep if wtfa_afd !=. // a good way to drop other merged observations

// The total cases we have is 8446, determined by the number of cases in the NHIS disability file

// to load the data into Stata but not run any analyses, select all the lines from .use comnhis through .keep if wtfa_afd !=.

// we describe key variables - mhisfib and f13 (shortcut) is the same
